# A Novel 'Transfibular Approach' for the Nonunion of a Tibial Shaft Fracture with Poor Anteromedial Soft Tissue Cover

**DOI:** 10.7759/cureus.315

**Published:** 2015-09-03

**Authors:** Raju Vaishya, Amit Kumar Agarwal, Harsh Singh, Vipul Vijay

**Affiliations:** 1 Orthopaedics, Indraprastha Apollo Hospital; 2 Orthopaedics, Indraprastha Apollo Hospitals

**Keywords:** tibial non-union, antero-medial scarring, transfibular approach

## Abstract

The need for an ideal approach for the nonunion of the tibial shaft with anteromedial soft tissue scarring has long baffled surgeons. Many different approaches have been suggested in the past, but all those approaches were haggled by a multitude of problems. We have described a novel 'transfibular approach' for this selective situation. An appropriate patient with a mid-shaft tibial non-union was selected. After preoperative workup, the patient underwent an open reduction internal fixation (ORIF) with lateral tibial plating, bone grafting, and partial fibulectomy. In this new approach, the plane between tibialis anterior and extensor hallucis longus was used combined with a conventional posterolateral approach using the same incision. Subsequently, the patient was followed up for adequacy of the fixation and wound-related problems with a convincing outcome.

## Introduction

The nonunion of a tibia with scarring over the anteromedial aspect of the leg is a challenging situation to manage through the standard approach [1]. The posterolateral approach has been described primarily for bone grafting in patients where infection of the tibia is either already present or is likely to occur [2]. Thus far, no unified approach has been described which enables lateral tibial plating, bone grafting, and partial fibulectomy through the same incision. Because of this, we describe a novel unified lateral (transfibular) approach for such cases with a non-united fracture of the tibial shaft and compromised anteromedial skin of the leg.

Informed patient consent was obtained for treatment of this patient.

**Figure 1:**
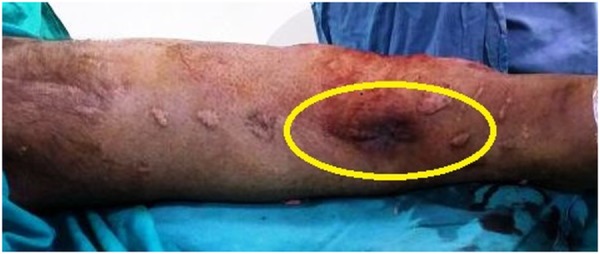
Clinical photograph of the leg showing scarred anteromedial skin over an ununited tibial fracture.

A 45-year-old patient had a road traffic accident six months back and sustained a compound Grade IIIB tibial fracture (Figure 1). A thorough debridement was done, and external fixation was applied. The anteromedial wound gradually healed by secondary intention. The external fixation was removed at the end of two months, and an interlocking nail was placed which became infected. The interlocked nail had to be removed and a debridement was carried out. The infection settled down after the debridement, and a below-knee slab was applied.

**Figure 2:**
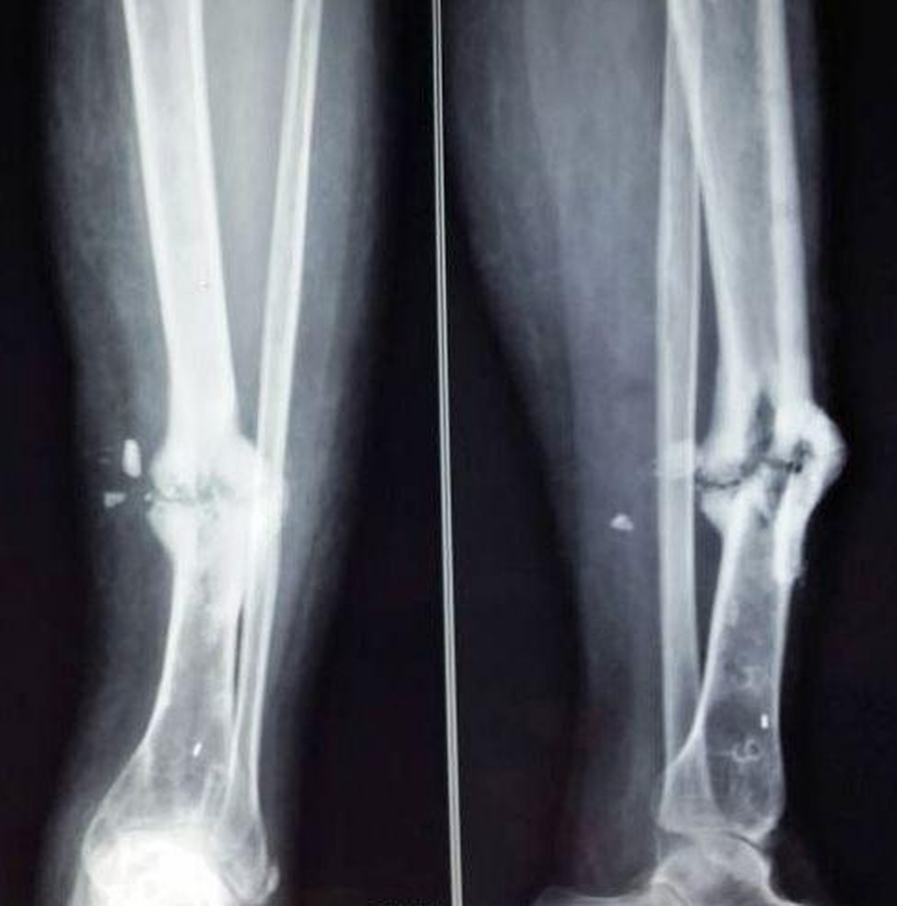
Preoperative radiographs showing nonunion of the tibia

At the end of six months, there was hypertrophic varus non-union at the tibial site that was apparent on the preoperative x-rays (Figure 2).

An open reduction and internal fixation (ORIF) of the tibia, bone grafting, and partial fibulectomy were planned, but due to a compromised anteromedial soft tissue cover, the standard approach could not be used. The interlocking nail was not intended at this time, as the fracture site has to be opened to freshen the bone ends to correct the varus deformity and to do bone grafting. Hence, ORIF with plating was the best option available in the given scenario. We used the novel unified lateral (transfibular) approach in this case and got convincing results.

## Technical report

**Figure 3:**
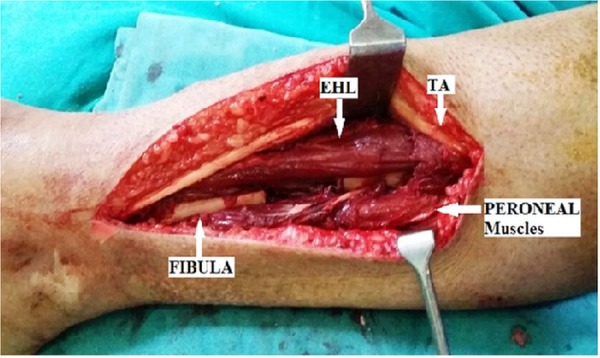
An internervous plane is developed between peroneal muscles anteriorly and soleus and flexor hallucis longus posteriorly.

An incision was made over the posterolateral border of the fibula to avoid the medial skin. Care was taken to protect the superficial peroneal nerve and short saphenous vein. An internervous plane was developed between the peroneal muscles anteriorly and the soleus and flexor hallucis longus posteriorly (Figure 3).

**Figure 4:**
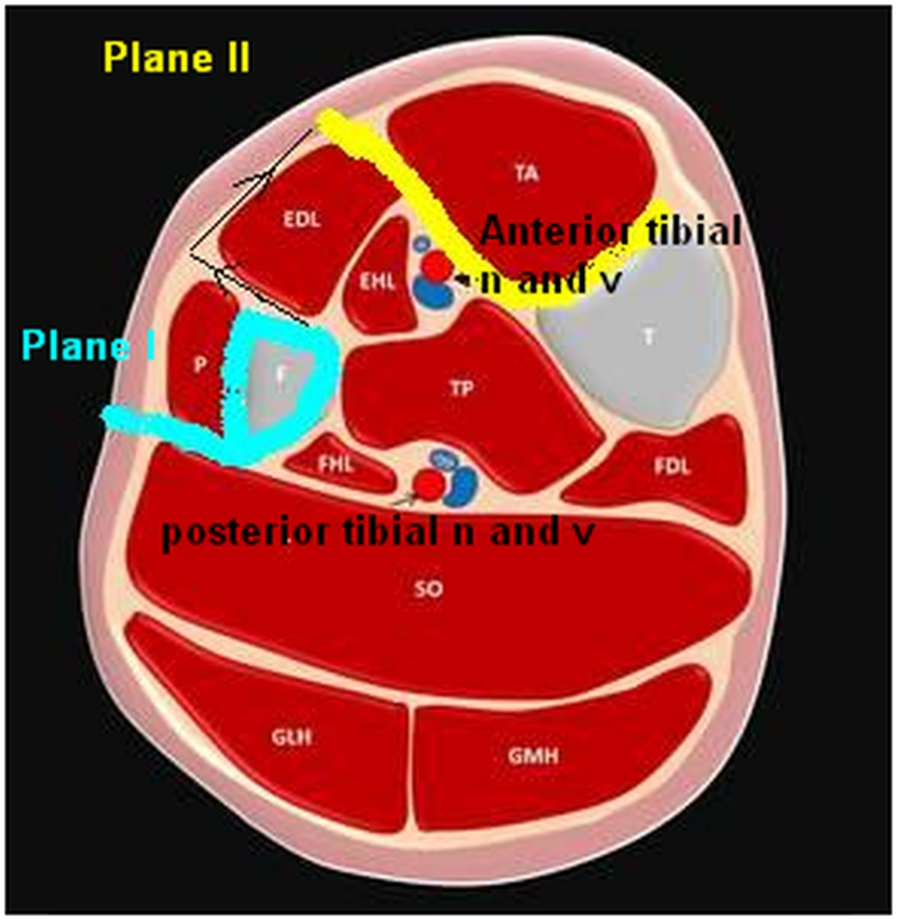
Cross sectional diagram of the middle third of the leg showing anatomy of the muscle planes and location of neurovascular bundles

The cross-sectional anatomy of the middle third of the leg should be very apparent to the surgeon for careful approaching through various muscle planes, and one has to be cautious about the location of neurovascular bundles (Figure 4).

**Figure 5:**
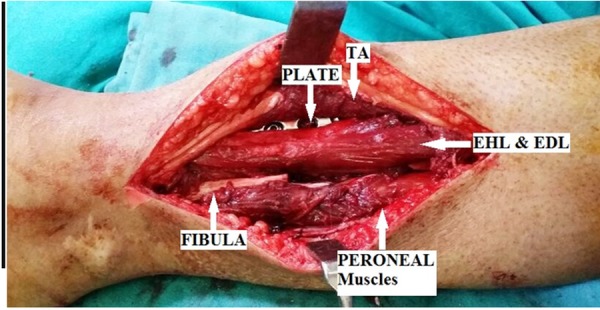
Lateral tibial plating with plane between tibialis anterior and extensor hallucis longus

The fibula was freed 2 cm above and below the nonunion site, and a 3 cm segment of the fibula was excised. The deep fascia overlying the muscles was cut, and another internervous plane was developed between tibialis anterior and extensor hallucis longus (Figure 5).

**Figure 6:**
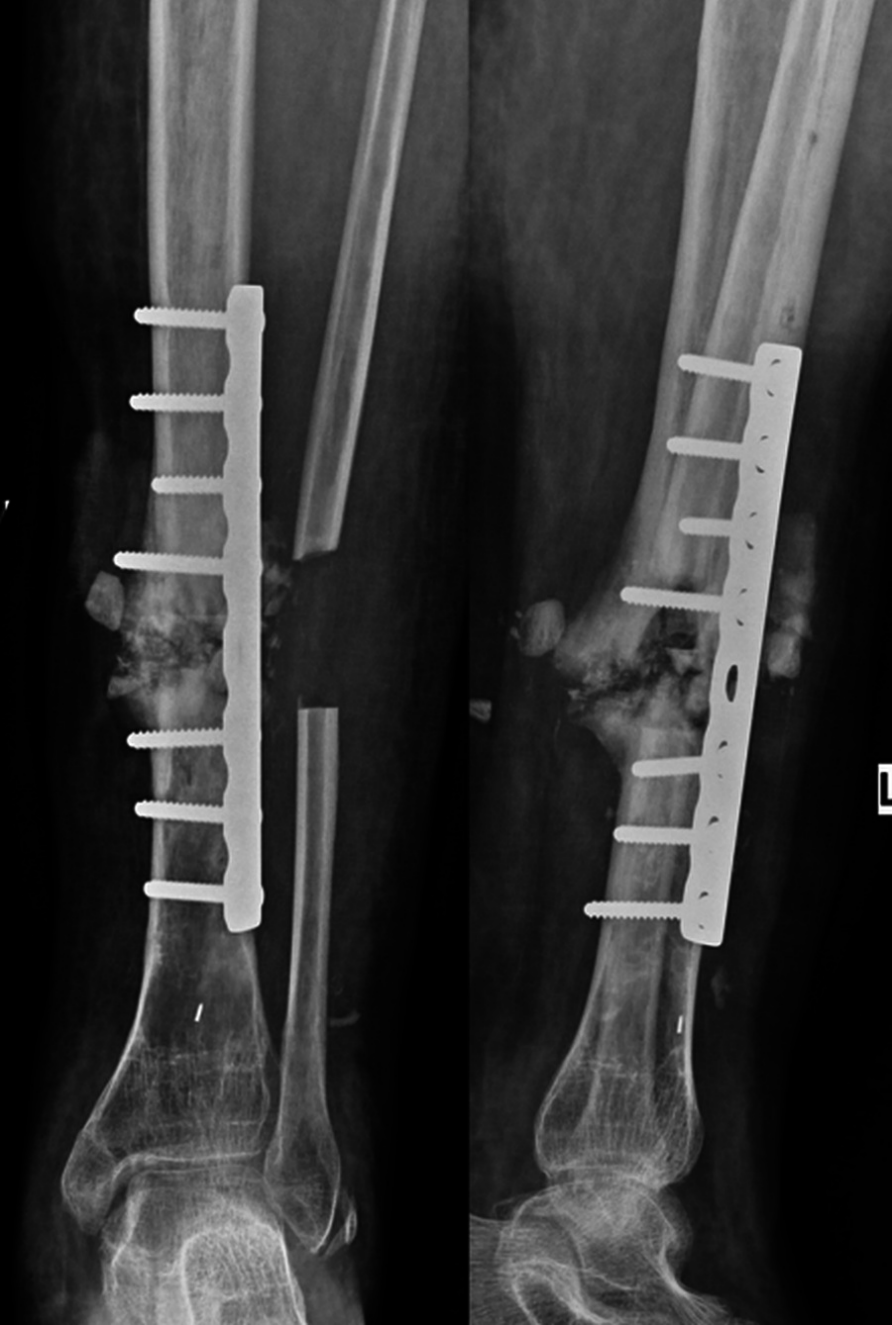
Postoperative radiographs showing lateral plating of the tibia, fibulectomy, and bone grafting.

A neurovascular bundle comprising the deep peroneal nerve and anterior tibial vessels along with the tibialis anterior muscle was retracted medially. The lateral aspect of the tibial shaft was then approached, and the periosteum overlying the lateral aspect was stripped. After meticulous clearing off of the fibrous tissue at the non-union site, a tibial plate was applied. The harvested fibula was used for bone grafting through the same incision over the posterolateral aspect of the non-union site. Postoperative follow-up x-ray of the leg was satisfactory (Figure 6).

## Discussion

Nonunion of the tibia with anteromedial soft tissue scarring is a challenging clinical problem. The management of these cases includes partial fibulectomy and ORIF with bone grafting for the nonunion tibia by avoiding compromised medial soft tissue. A transfibular approach is an excellent approach for the nonunion of a tibial shaft with anteromedial scarring as multiple procedures can be carried out through the same single incision. The other advantage of this approach is that the anterior neurovascular structures are directly visualized and protected.

Some surgeons prefer to use a double incision approach using an anterior and a posterolateral incision, but problems with wound closure and early wound dehiscence are common in such settings. Jones, et al. [3] propounded that grafting through the posterolateral approach is considered helpful in patients with infection of the tibia or in whom it is likely to occur [4]. Certain other approaches have been described using an anterolateral approach and developing a plane between peroneus muscles and extensor digitorium longus. However, through such a plane, it is practically difficult to approach the lateral tibial border for plating [5]. The present approach does away with this problem and allows for easy and safe exposure of lateral tibial cortex, at the same time permitting bone grafting and partial fibulectomy.

The importance of uncompromised blood flow in wound healing is crucial [6]. It is well understood now that an anterior approach in the above circumstances would have invariably resulted in undue stress over the wound site due to tense wound margins after closure. Moreover, the scarred tissue would have difficulty with primary healing. With the use of the standard posterolateral approach, other problems arise, especially when the plane has to be extended distally. The risk to the peroneal artery cannot be overemphasised when dissecting in the posterior compartment through the posterolateral approach. Caution is advised as the bifurcation of the perforating artery may be as little as 41 mm from the tibial plafond. This is important during deep dissection when the belly of the flexor hallucis longus muscle is reflected medially from the medial edge of the fibula [7]. Thus, a combined approach as described above makes it less mutilating in the posterior compartment and, hence, provides a relatively safer window.

## Conclusions

The transfibular approach can be utilized for multiple procedures required to treat a nonunion tibial shaft in a selective case with compromised anteromedial soft tissue cover. The various procedures, which can be done through the same skin incision but by making different muscle planes, are open reduction internal fixation with tibial plating, bone grafting, and fibulectomy.
